# Supplementary material for: Alteration of RNA m6A methylation mediates aberrant RNA binding protein expression and alternative splicing in condyloma acuminatum
Source: PeerJ. 2024 May 20;12:e17376. doi: 10.7717/peerj.17376 (PMC11114121; doi:10.7717/peerj.17376)
Supplement: Supplemental Information 1 [file peerj-12-17376-s001.docx]

**Supplementary material: RT-qPCR process**

1. **RNA extraction and quality detection**

Total RNA was extracted and isolated using RNAisoTM PLUS (TaKaRa, Japan) under the RNase free condition following the manufacturer’s protocol. Nanodrop Lite (Thermofisher, USA) was used to detect the quality and quantity of RNAs by the absorbance at 260 nm/280 nm (A260/A280). Denaturing agarose gel electrophoresis was used to detect RNA purity and integrity.

1. **Reverse transcription**

PrimeScript™ RT reagent Kit with gDNA Eraser (TaKaRa, Japan) was used for erasing genomic DNA and RNA transcription, with the reaction volume of 10 µl (reaction system 1) and 20 µl (reaction system 2) and the PCR reactions as below, respectively.

| Reaction system (1) | Volume | PCR reaction |
| --- | --- | --- |
| 5×gDNA Eraser Buffer | 2 µl |  |
| gDNA Eraser | 1 µl | 42 ℃ 2 minutes |
| Total RNA | 1 µg | 4 ℃ ∞ |
| RNase free H_2_O | Up to 10 µl |  |

| Reaction system (2) | Volume | PCR reaction |
| --- | --- | --- |
| 5× Buffer | 2 µl |  |
| RT Enzyme Mix | 1 µl | 37 ℃ 15 minutes |
| Mix I | 10 µl | 85 ℃ 5 seconds |
| Primer | 1 µl | 4 ℃ ∞ |
| RNase free H_2_O | 6 µl |  |
| Total volume | 20 µl |  |

**Real-time PCR**

RT‒qPCR was performed using the ABI Prism 7900 (Applied Biosystems, Foster City, CA, USA) and SYBR PrimeScript RT-PCR kit II (TaKaRa, Japan) according to the manufacturer’s instructions. Primer sequences of target DEGs were designed and synthesized by RiboBio (Guangzhou, China) and are shown in Tab. S2. The specificity of the primer pair was verified by Primer-BLAST ([https://www.ncbi.nlm.nih.gov/tools/primer-blast/](https://www.ncbi.nlm.nih.gov/tools/primer-blast/.)). GAPDH was used as a reference gene. The PCR conditions were as follows: denaturing for 10 minutes at 95 ℃, then 40 cycles including denaturation for 15 seconds at 95 ℃, with annealing and extension for 1 minute at 60 ℃. Three technique replicates were performed for PCR amplification of each sample.

| Reaction system (3) | Volumn |
| --- | --- |
| 2× SYBR@ Premix Ex TaqII | 10 µl |
| F Primer(10µM) | 0.8 µl |
| R Primer(10µM) | 0.8 µl |
| ROX Reference II | 0.4 µl |
| cDNA template | 2 µl |
| ddH_2_O | 6 µl |
| Total volume | 20 µl |

1. **Validation of qPCR**

The specificity of PCR products was detected by 1.5% agarose nucleic acid electrophoresis. NTCs were used to detect PCR contamination in SYBR Green I reactions. NTCs were included in each plate, and the Cq of NTCs ≥ 40 were ignored if the maximum value of Cq is 35. At the same time, a positive control of nucleic acid was used to monitor changes in the experiment over time. For linear dynamic range, Cq variation was set as 12 at lower limit and 35 for the limitation of detection. Outliers were identified and indicated by instrument cue and we repeated the RT-PCR for the samples.

1. **Statistical analysis**

The Cq data were collected automatically. The relative expression for a target gene was calculated using 2^-△△Cq^. △Cq was calculated by △Cq = Cq of target gene - Cq of GAPDH, and -△△Cq was calculated by -△△Cq = average △Cq of the CON group - △Cq of the CA group. AS ratio was calculated by AS ratio = the relative expression of gene-AS / gene-M. Quantitative variables were expressed as means ± standard error (SE). All calculations and plots were completed using GraphPad v9. Student’s t test was used to compare the expression of target genes and AS ratio changes for target gene splicing events. A *P* value < 0.05 was considered statistically significant.
