# Supplementary material for: Alteration of RNA m6A methylation mediates aberrant RNA binding protein expression and alternative splicing in condyloma acuminatum
Source: PeerJ. 2024 May 20;12:e17376. doi: 10.7717/peerj.17376 (PMC11114121; doi:10.7717/peerj.17376)
Supplement: Table S1 [file peerj-12-17376-s003.docx]

| **Table S1. Characteristics of the study subjects** | | | | |
| --- | --- | --- | --- | --- |
| Characteristics | | CA group (n=40) | CON Group (n=15) | *P* value |
| Ages (Mean, SD) | | 26.63 ±4.91 | 25.35 ± 5.10 | 0.398 |
| BMI (Mean, SD) | | 21.58 ± 0.76 | 21.23 ± 0.64 | 0.119 |
| HPV genotyping (n) | Single low-risk | 8 | 0 |  |
|  | With HPV 16/18 | 9 | 0 |  |
|  | With other high-risk | 23 | 0 |  |
| Disease courses at first visit | < 6 months | 25 | / |  |
|  | 6-12 months | 9 | / |  |
|  | ≥ 12 months | 6 | / |  |
