# Supplementary material for: Alteration of RNA m6A methylation mediates aberrant RNA binding protein expression and alternative splicing in condyloma acuminatum
Source: PeerJ. 2024 May 20;12:e17376. doi: 10.7717/peerj.17376 (PMC11114121; doi:10.7717/peerj.17376)
Supplement: Table S2 [file peerj-12-17376-s004.docx]

| **Table S2. Primer sequences for target genes** | |
| --- | --- |
| DNMT1-F | TGAACGGACAGATTGACAT |
| DNMT1-R | AGGAAGCTGCTAAGGACTA |
| ZC3H12D -F | GGTGGAGATGGAGTGGAA |
| ZC3H12D -R | GGATAGGGATGGGAAATGAG |
| DZIP1L-F | CTGGTGACTTCCTTGACAA |
| DZIP1L-R | CGATTCAGACTCTCAGACA |
| RASAL2-M-F | ACTAAACTGGAGGTACCAGCA |
| RASAL2-AS-F | CAGACCCCAGAGGTACCAGCA |
| RASAL2-M/AS-R | TTTGAAGGGTGAGGTATTTG |
| YWHAZ –M/AS-F | ACCTACGGGCTCCTACAACA |
| YWHAZ -AS-R | AGTGGCGCAGAACATCCAGT |
| YWHAZ -M-R | GAGATAAAAAGAACATCCAGT |
