# Supplementary material for: Alteration of RNA m6A methylation mediates aberrant RNA binding protein expression and alternative splicing in condyloma acuminatum
Source: PeerJ. 2024 May 20;12:e17376. doi: 10.7717/peerj.17376 (PMC11114121; doi:10.7717/peerj.17376)
Supplement: Table S3 [file peerj-12-17376-s005.docx]

| **Table S3. MeRIP-seq generated in this study** | | | | | | |
| --- | --- | --- | --- | --- | --- | --- |
| **SampleID** | **clean_**  **total** | **clean_**  **base** | **Total mapped** | **Total Uniquely mapped** | **Splice reads** | **Nonsplice reads** |
| CA_IP_1 | 73611640 | 9.96G | 69380096  (94.25%) | 67258953  (96.94%) | 6982085  (10.38%) | 60276868  (89.62%) |
| CA_IP_2 | 84765698 | 11.50G | 79279021  (93.53%) | 77050162  (97.19%) | 6455121  (8.38%) | 70595041  (91.62%) |
| CA_IP_3 | 77157204 | 10.44G | 72287839  (93.69%) | 69976403  (96.80%) | 5381348  (7.69%) | 64595055  (92.31%) |
| CA_IP_4 | 83813130 | 11.29G | 76645678  (91.45%) | 74323643  (96.97%) | 6106581  (8.22%) | 68217062  (91.78%) |
| CA_IP_5 | 87929196 | 11.86G | 81289586  (92.45%) | 78911723  (97.07%) | 5630286  (7.13%) | 73281437  (92.87%) |
| CA_input_1 | 89333786 | 8.93G | 84264900  (94.33%) | 80871011  (95.97%) | 8637786  (10.68%) | 72233225  (89.32%) |
| CA_input_2 | 92505376 | 9.25G | 88768200  (95.96%) | 85233733  (96.02%) | 9464731  (11.10%) | 75769002  (88.90%) |
| CA_input_3 | 91851274 | 9.19G | 86972194  (94.69%) | 83435812  (95.93%) | 8052003  (9.65%) | 75383809  (90.35%) |
| CA_input_4 | 87582188 | 8.76G | 83377297  (95.20%) | 79204827  (95.00%) | 9367150  (11.83%) | 69837677  (88.17%) |
| CA_input_5 | 93734496 | 9.37G | 89442559  (95.42%) | 86116675  (96.28%) | 8250761  (9.58%) | 77865914  (90.42%) |
| CON_IP_1 | 80159626 | 10.87G | 75116464  (93.71%) | 72725386  (96.82%) | 6117231  (8.41%) | 66608155  (91.59%) |
| CON_IP_2 | 80073884 | 10.86G | 75079440  (93.76%) | 72725583  (96.86%) | 6744054  (9.27%) | 65981529  (90.73%) |
| CON_IP_3 | 88414204 | 11.63G | 82994256  (93.87%) | 79408888  (95.68%) | 9373005  (11.80%) | 70035883  (88.20%) |
| CON_IP_4 | 89052664 | 12.06G | 84413195  (94.79%) | 81741812  (96.84%) | 6749959  (8.26%) | 74991853  (91.74%) |
| CON_IP_5 | 73136162 | 9.87G | 69286916  (94.74%) | 67041530  (96.76%) | 6367190  (9.50%) | 60674340  (90.50%) |
| CON_input_1 | 82532490 | 8.25G | 78199887  (94.75%) | 74995995  (95.90%) | 7145850  (9.53%) | 67850145  (90.47%) |
| CON_input_2 | 85485796 | 8.55G | 81071331  (94.84%) | 78124820  (96.37%) | 8167836  (10.45%) | 69956984  (89.55%) |
| CON_input_3 | 90764520 | 9.08G | 85264962  (93.94%) | 82137567  (96.33%) | 9234920  (11.24%) | 72902647  (88.76%) |
| CON_input_4 | 85815702 | 8.58G | 80796377  (94.15%) | 77978956  (96.51%) | 6768330  (8.68%) | 71210626  (91.32%) |
| CON_input_5 | 88984772 | 8.90G | 83656065  (94.01%) | 80126807  (95.78%) | 8359366  (10.43%) | 71767441  (89.57%) |
