# Supplementary figures and images for: Alteration of RNA m6A methylation mediates aberrant RNA binding protein expression and alternative splicing in condyloma acuminatum
Source: PeerJ. 2024 May 20;12:e17376. doi: 10.7717/peerj.17376 (PMC11114121; doi:10.7717/peerj.17376)

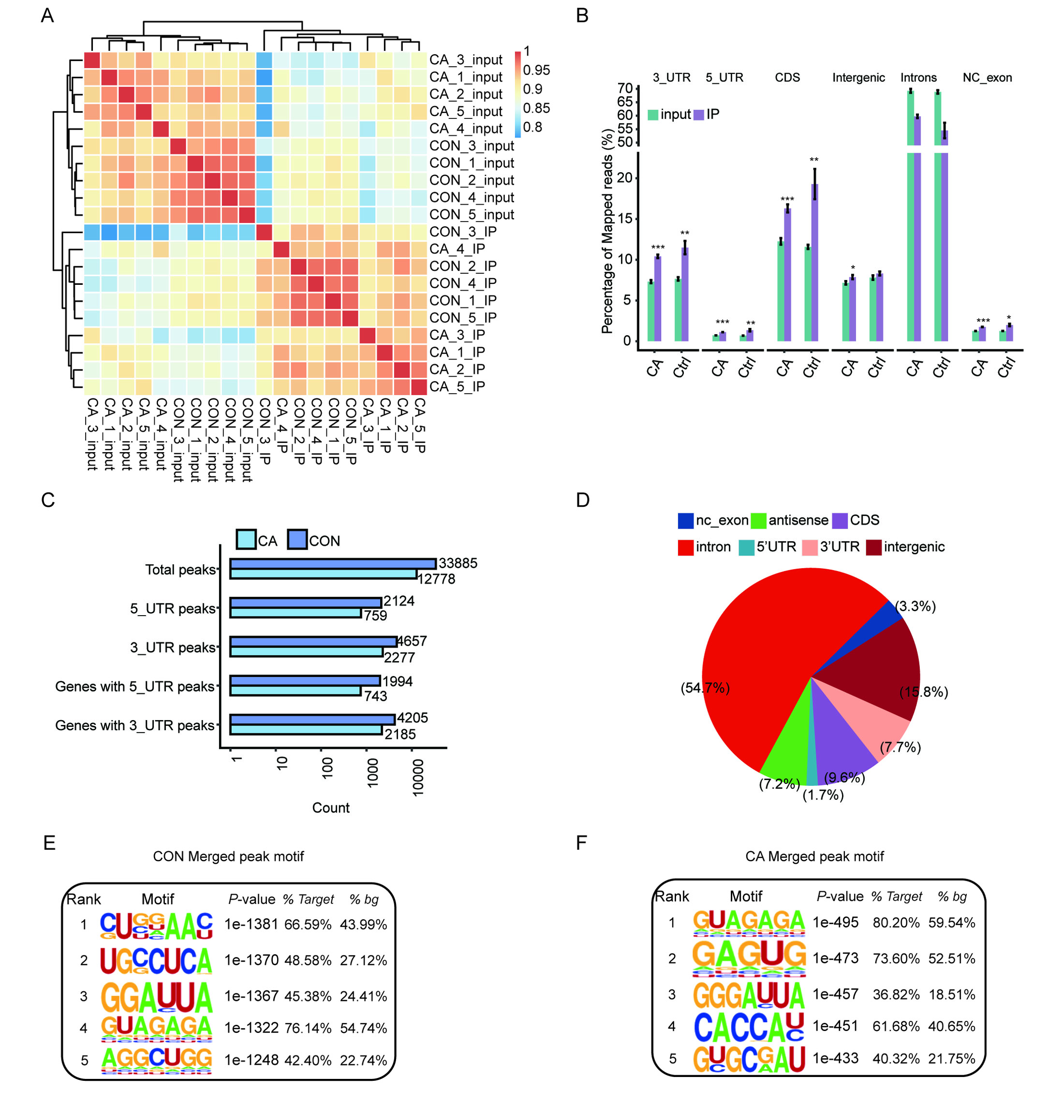

Supplement: Figure S1 — (A) Heatmap clustering analysis of sample correlation based on the normalized mapped reads on each gene, showing immunoprecipitated samples clustered.(B) Read distribution of IP and input samples in different genome regions. All qualified IP (n=10) and input (n=10) samples 0.01 and *P < 0.05, ** P ¡ 0.01, ***P < 0.001. (C) The number of peaks and genes with m6A peaks detected in at least 3 CA or CON samples. (D) The distribution of identified m6A peaks in different gene regions. (E) The top five enriched motif in the m6A peaks detected by HOMER in CON samples. (F) The top five enriched motif in the m6A peaks detected by HOMER in CA samples. [file peerj-12-17376-s006.png]

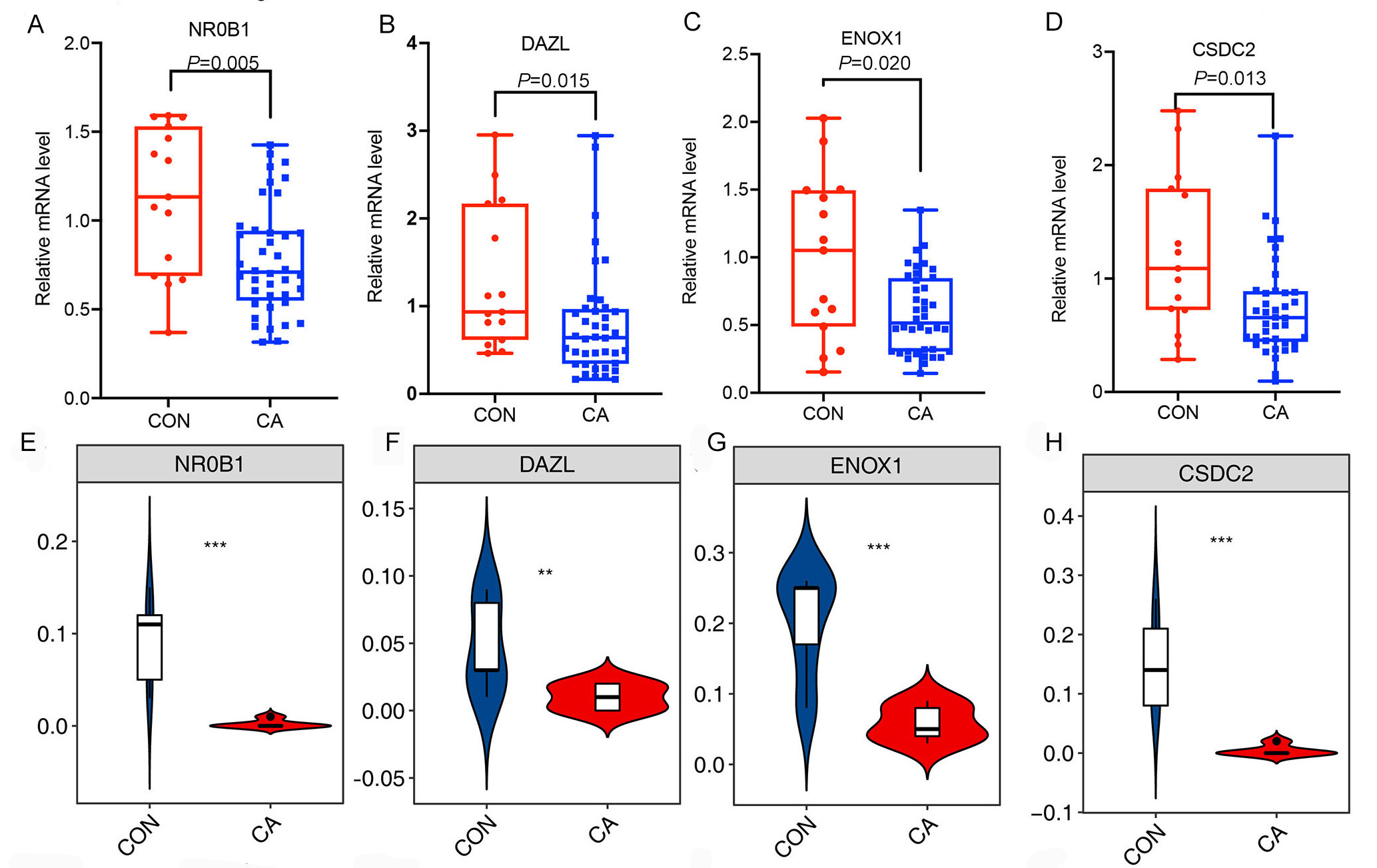

Supplement: Figure S2 — (A-D)Bar plot showing the relative expression of NR0B1, DAZL, ENOX1 and CSDC2 in additional CON and CA tissue samples using RT-qPCR. (E-H) Violin plot showing the FPKM expression of DMNT1, ZC3H12D and DZIP1L using RNA-sequencing. *, P <0.05; **, P <0.01; ***, P <0.001. [file peerj-12-17376-s007.png]

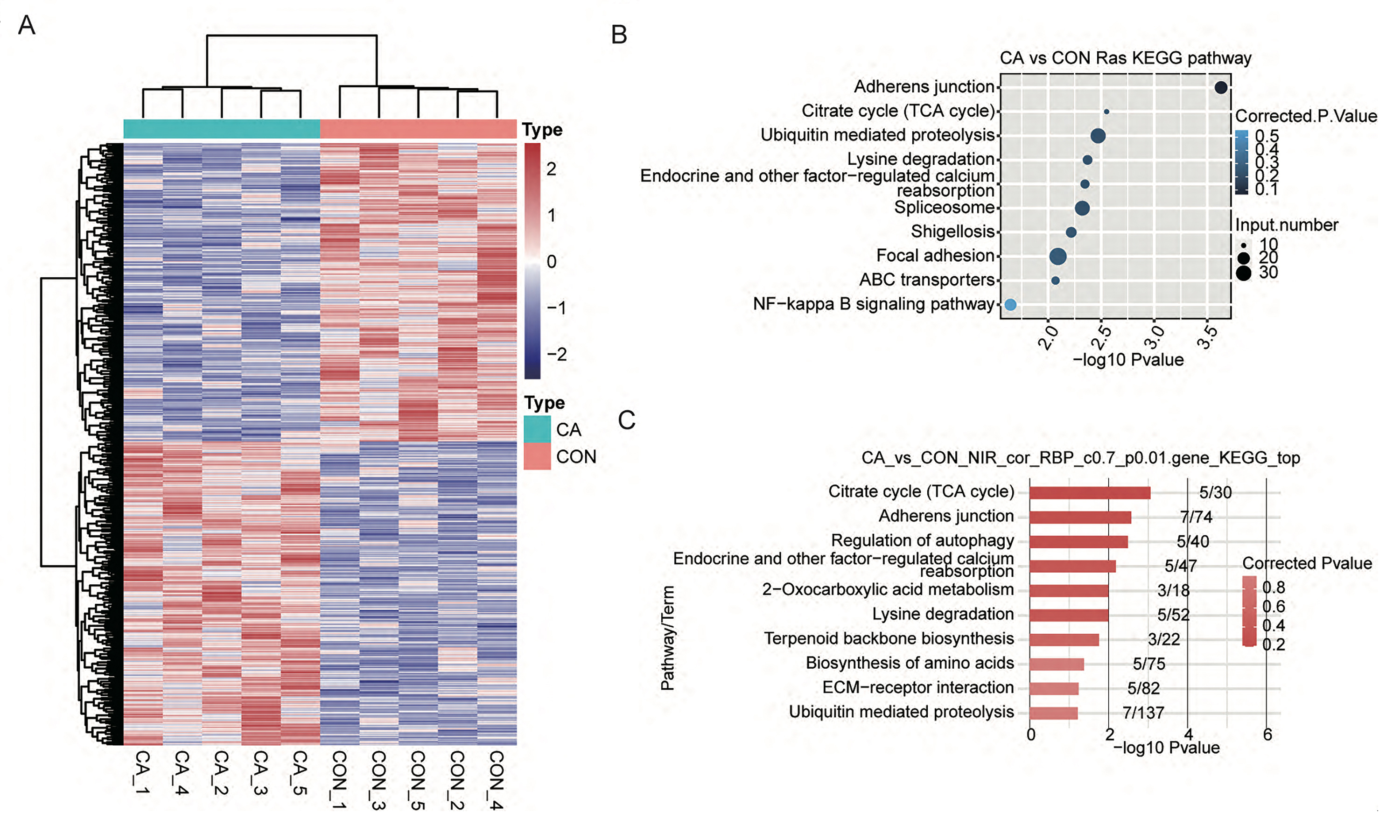

Supplement: Figure S3 — (A) Hierarchical clustering heatmap showing the PSI values of all RASEs. (B) Bubble plot showing the top 10 enriched KEGG pathways for RASGs in CA vs. CON. (C) Bar plot exhibiting the most enriched KEGG pathways that were illustrated for RASGs codisturbed by specific RBPs-associated m6A in CON or CA samples. [file peerj-12-17376-s008.png]

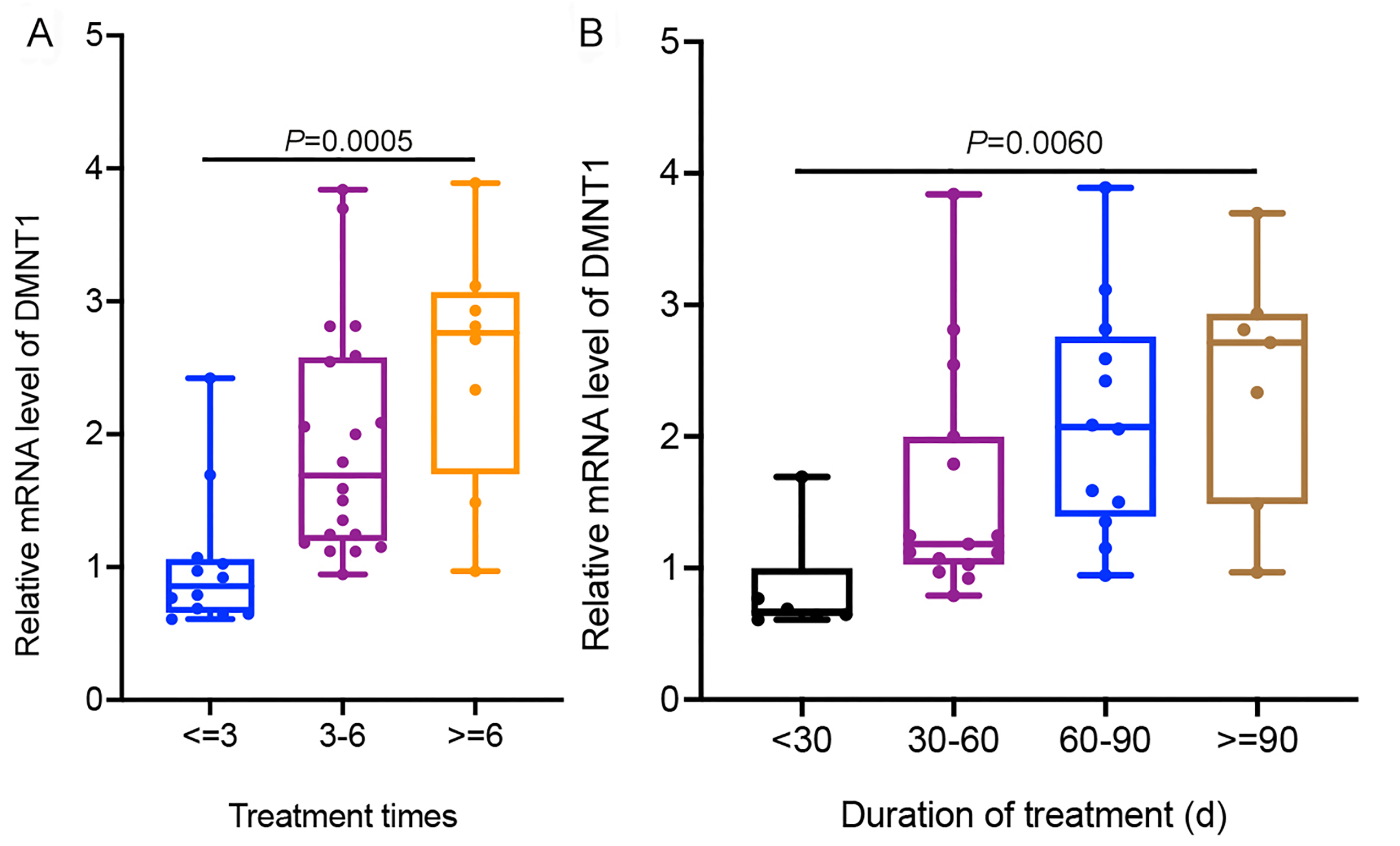

Supplement: Figure S4 — (A) The relative mRNA expression of DMNT1 in groups with viable treatment times in CA. (B) The relative mRNA expression of DMNT1 in groups with viable duration of treatment in CA. [file peerj-12-17376-s009.png]
